# Elevated expression of MMP-2 and TIMP-2 cooperatively correlates with risk of lung cancer

**DOI:** 10.18632/oncotarget.20156

**Published:** 2017-08-11

**Authors:** Chao Cao, Ning Xu, Xiaoxia Zheng, Wenxue Zhang, Tianwen Lai, Zaichun Deng, Xiaoping Huang

**Affiliations:** ^1^ Department of Respiratory Medicine, Ningbo First Hospital, Ningbo, China; ^2^ Department of Respiratory, Institute of Respiratory Diseases, The Affiliated Hospital of Guangdong Medical University, Zhanjiang, China; ^3^ Department of Respiratory Medicine, Affiliated Hospital, Ningbo University School of Medicine, Ningbo, China

**Keywords:** MMP-2, TIMP-2, lung cancer, diagnosis, biomarker

## Abstract

Lung cancer is one of the most common form of malignant diseases and the leading cause of cancer-related mortality worldwide. It is reported that approximately two-thirds of lung cancer patients is the presence of advance disease at the time of diagnosis. Hence novel lung cancer diagnostic tests, which can be used to screen individuals at high risk, are required. In the derivation cohort, a total of 88 patients admitted into hospital with suspected lung cancer were included. Bronchial alveolar lavage fluid (BALF) and lung tissue samples were collected from included patients, and were analyzed for MMP-2 and TIMP-2 expression. The results showed a higher level of MMP-2 and TIMP-2 expression and secretion in airways of lung cancer patients than that of benign diseases. A statistically significant correlation was observed between MMP-2 and TIMP-2. In addition, a validation cohort involving 107 patients was conducted to confirm these results. Interesting, BALF MMP-2 and TIMP-2 showed a high sensitivity and specificity in predicting the malignant nature of pulmonary disease in both derivation cohort and validation cohort. The findings in this study suggested that elevated expression of MMP-2 and TIMP-2 cooperatively correlates with risk of lung cancer. Measurement of MMP-2 and TIMP-2 in BALF might be helpful for differential diagnosis of primary lung cancer.

## INTRODUCTION

Lung cancer accounts for nearly one fifth of the cancer related deaths worldwide [1]. Although numbers of new technologies and tools were applied in recent years, there are still more than 75% of cases are diagnosed at a late stage not amenable to surgery [2]. It is reported that 5-year survival rate is only 15% for patients diagnosed with lung cancer in the advanced stage [3]. Thus, there is a great need to identify lung cancer at an early stage, ideally before cancer cells invasion and metastasis. This has led to significant interest in high sensitivity and specificity screening methods to detect early-stage cancers, particularly for individuals with high risk.

Understanding of the biological pathways in the development of lung cancer is crucial to identify key biomolecules that could be of significant clinical value. Matrix metalloproteinases (MMPs) are zinc-dependent proteases that play a major role in extracellular matrix (ECM) remodeling but have also been shown to be involved in the regulation of multiple stages of cancer progression [4]. So far, more than 20 members of the MMP family were identified, among which MMP-2 has been extensively studied in human cancers and has been shown to be closely related to the invasive potential and metastasis of different types of tumor cells including lung cancer [5–7]. However, the expression of MMP-2 and its inhibitor the tissue inhibitors of matrix metalloproteinase 2 (TIMP-2) in airways of lung cancer and its diagnostic value is still unclear. We therefore performed the present study to evaluate the expression of MMP-2 and TIMP-2 in airways of lung cancer patients by comparing levels of them in benign diseases.

## RESULTS

### Patient characteristics

Basic characteristics and tumor characteristics for patients in the derivation cohort and the validation cohort were summarized in Table 1 and Supplementary Table 1, respectively. In the derivation cohort, there were 48 lung cancer patients and 40 patients with benign diseases. The pathologic types included 18 squamous cell carcinomas, 18 adenocarcinomas, and 12 small cell carcinomas. There were 27 patients with pneumonia, 9 patients with inflammatory nodules, 3 with tuberculosis, and 1 with pulmonary sarcoidosis in the control group. In the validation cohort, there were 41 lung cancer patients and 66 patients with benign diseases. The pathologic types included 19 squamous cell carcinomas (SCC), 14 adenocarcinomas (ADC), and 8 small cell carcinomas (SCLC). There were 57 patients with pneumonia, 7 patients with inflammatory nodules, 2 with tuberculosis in the control group. In addition, 20 lung tissue samples were collected for immunohistochemistry (IHC) study: 4 squamous cell carcinomas, 3 adenocarcinomas, 3 small cell carcinomas, 7 pneumonia, and 3 inflammatory nodules.

**Table 1:** Clinical characteristics of patients with lung cancer and benign diseases

	Lung cancer (*n* = 48)	Benign diseases (*n* = 40)	*P* value
Age, years			0.288
Mean ± SEM	62.7 ± 1.4	60.7 ± 1.3	
Gender, *n* (%)			0.329
Male	39 (81.3%)	29 (72.5%)	
Female	9 (18.7%)	11 (27.5%)	
Smoking status, *n* (%)			0.842
Smokers	29 (60.4%)	25 (62.5%)	
Non-smokers	19 (39.6%)	15 (37.5%)	
Pack-years	49.8 ± 6.5	45.4 ± 6.0	0.732
Cell type, *n* (%)			
Squamous cell carcinoma	18 (37.5%)		
Adenocarcinoma	18 (37.5%)		
Small-cell lung cancer	12 (25.0%)		
TNM stage, *n* (%)			
I	8 (16.7%)		
II	14 (29.2%)		
III	10 (20.8%)		
IV	16 (33.3%)		

### The expression of MMP-2 in lung cancer patients and benign diseases

**Figure 1:**
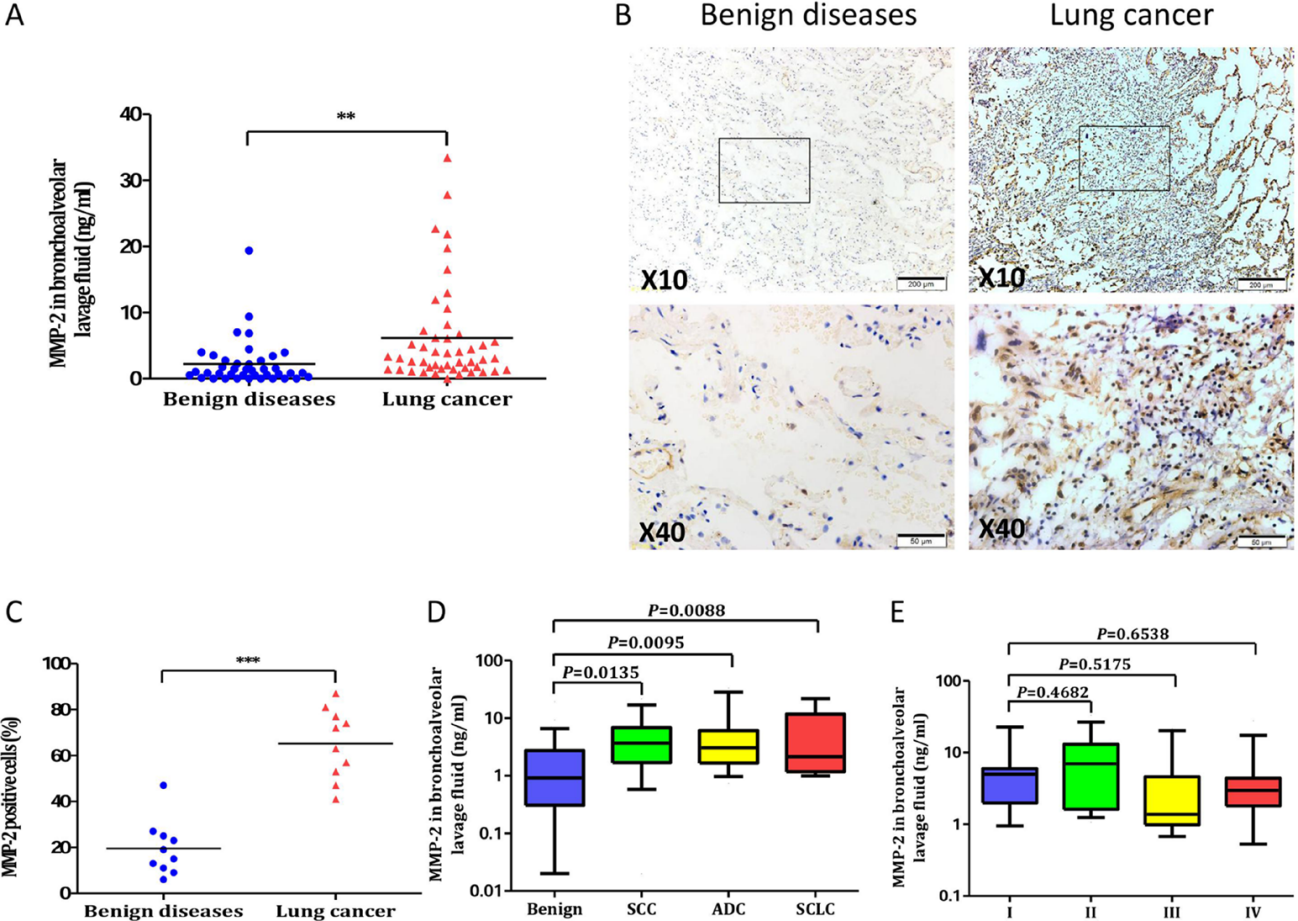
MMP-2 in lung cancer patients and benign diseases (**A**) Comparison of MMP-2 levels in BALF between lung cancer and control group. The levels of MMP-2 were significantly higher in lung cancer patients than those in controls (**P < 0.01). (**B**) Representative examples of immunohistochemistry of MMP-2 expression in human lung specimens. (**C**) Quantification of MMP-2 expression in the lung cancer and benign diseases (***P < 0.001). (**D**) In subgroup analysis by tumor histology, BALF MMP-2 remained significantly higher in different cell types of lung cancer patients than benign controls. (**E**) BALF MMP-2 concentration were assessed according to TNM stage. Values in the box plot are given as median (interquartile range); BALF, bronchoalveolar lavage fluid; SCC, squamous cell carcinoma; ADC, adenocarcinoma; SCLC, small-cell lung cancer.

The levels of MMP-2 was detected in the Bronchial alveolar lavage fluid (BALF) of 48 lung cancer patients and 40 benign diseases. As shown in Figure 1A, a significant higher MMP-2 levels was observed among patients with lung cancer than benign diseases (6.1 ± 1.1 ng/ml *versus* 2.2 ± 0.6 ng/ml, *P* = 0.0033). The expression of MMP-2 in lung tissues was further analyzed by IHC staining (Figure 1B). The percentage of MMP-2 positive cells in lung cancer patients was significant higher than that of benign group (65.2% ± 4.9% *versus* 19.5 ± 3.8%, *P* < 0.0001; Figure 1C). The levels of MMP-2 in BALF from 48 lung cancer patients were analyzed according to tumor histology. A statistically significant difference was found between patients with different cell type of lung cancer and patients with benign diseases (5.4 ± 1.4 ng/ml for SCC, *P* = 0.0135; 6.7 ± 2.2 ng/ml for ADC, *P* = 0.0095; 6.5 ± 2.2 ng/ml for SCLC, *P* = 0.0088; Figure 1D). However, BALF MMP-2 concentration were not associated with TNM stage (Figure 1E).

### The expression of TIMP-2 in lung cancer patients and benign diseases

**Figure 2:**
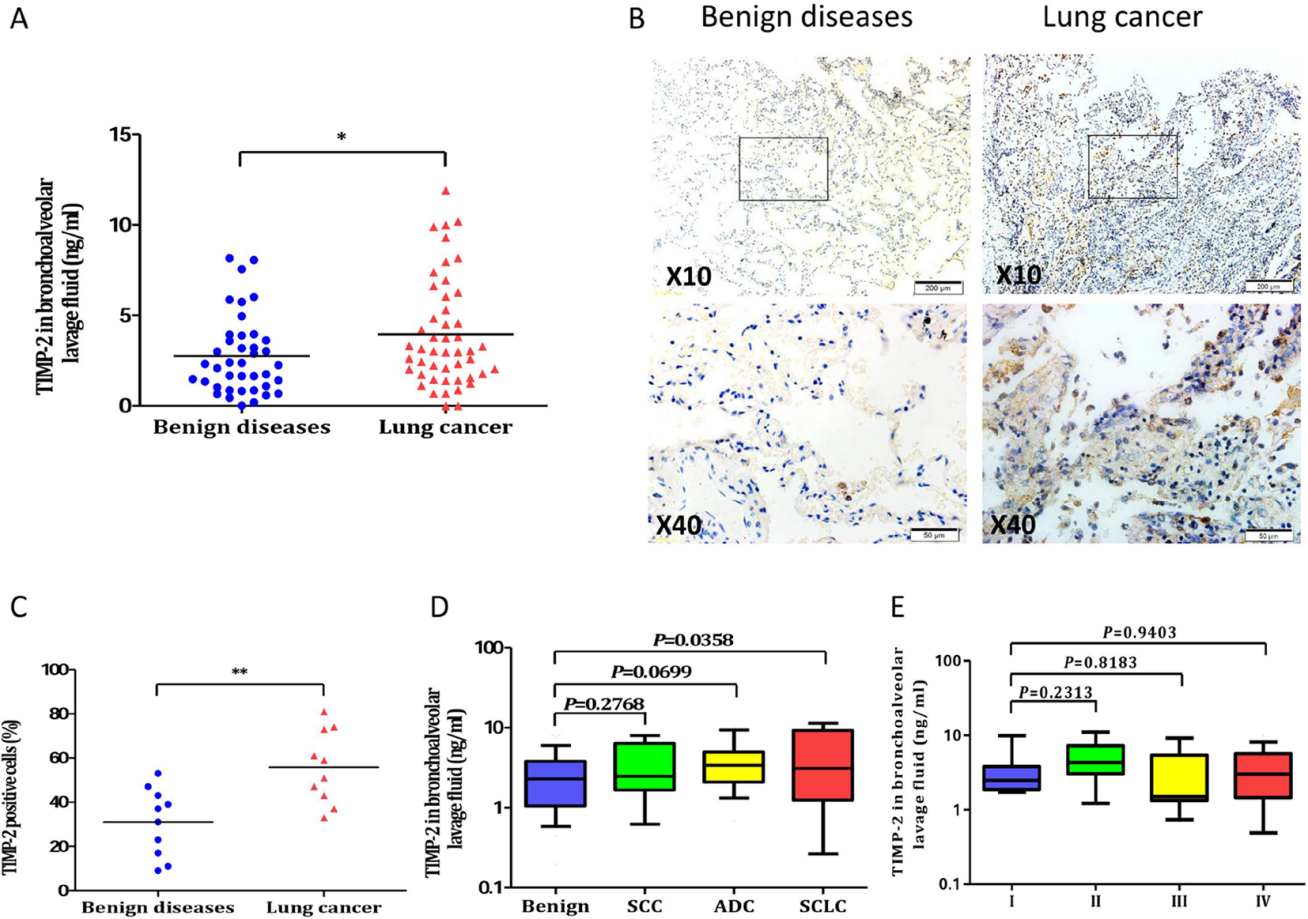
TIMP-2 in lung cancer patients and benign diseases (**A**) Comparison of TIMP-2 levels in BALF between lung cancer and control group. The levels of TIMP-2 were significantly higher in lung cancer patients than those in controls (*P < 0.05). (**B**) Representative examples of immunohistochemistry of TIMP-2 expression in human lung specimens. (**C**) Quantification of TIMP-2 expression in the lung cancer and benign diseases (**P < 0.01). (**D**) In subgroup analysis by tumor histology, BALF MMP-2 in different cell types of lung cancer patient. (**E**) BALF TIMP-2 concentration were assessed according to TNM stage. Values in the box plot are given as median (interquartile range); BALF, bronchoalveolar lavage fluid; SCC, squamous cell carcinoma; ADC, adenocarcinoma; SCLC, small-cell lung cancer.

The level of BALF TIMP-2 was significant higher among patients with lung cancer than patients with benign diseases (4.0 ± 0.4 ng/ml *versus* 2.8 ± 0.3 ng/ml, *P* = 0.0371; Figure 2A). A higher expression of TIMP-2 were also observed in IHC staining (Figure 2B). The percentage of TIMP-2 positive cells in lung cancer patients was significant higher than that of benign group (55.9% ± 5.2% *versus* 31.0 ± 4.9%, *P* = 0.0026; Figure 2C). When the lung cancer cases were categorized by tumor histology, however, a statistically significant difference was found between patients with small cell carcinoma and patients with benign diseases (4.7 ± 1.2 ng/ml *versus* 2.8 ± 0.3 ng/ml, *P* = 0.0358; Figure 2D), while no significant difference was found between malignant and benign groups with respect to adenocarcinoma or squamous cell carcinoma (data not shown). Moreover, BALF TIMP-2 concentration were not associated with TNM stage (Figure 2E).

### The relationship between smoking, MMP-2, and TIMP-2

**Figure 3:**
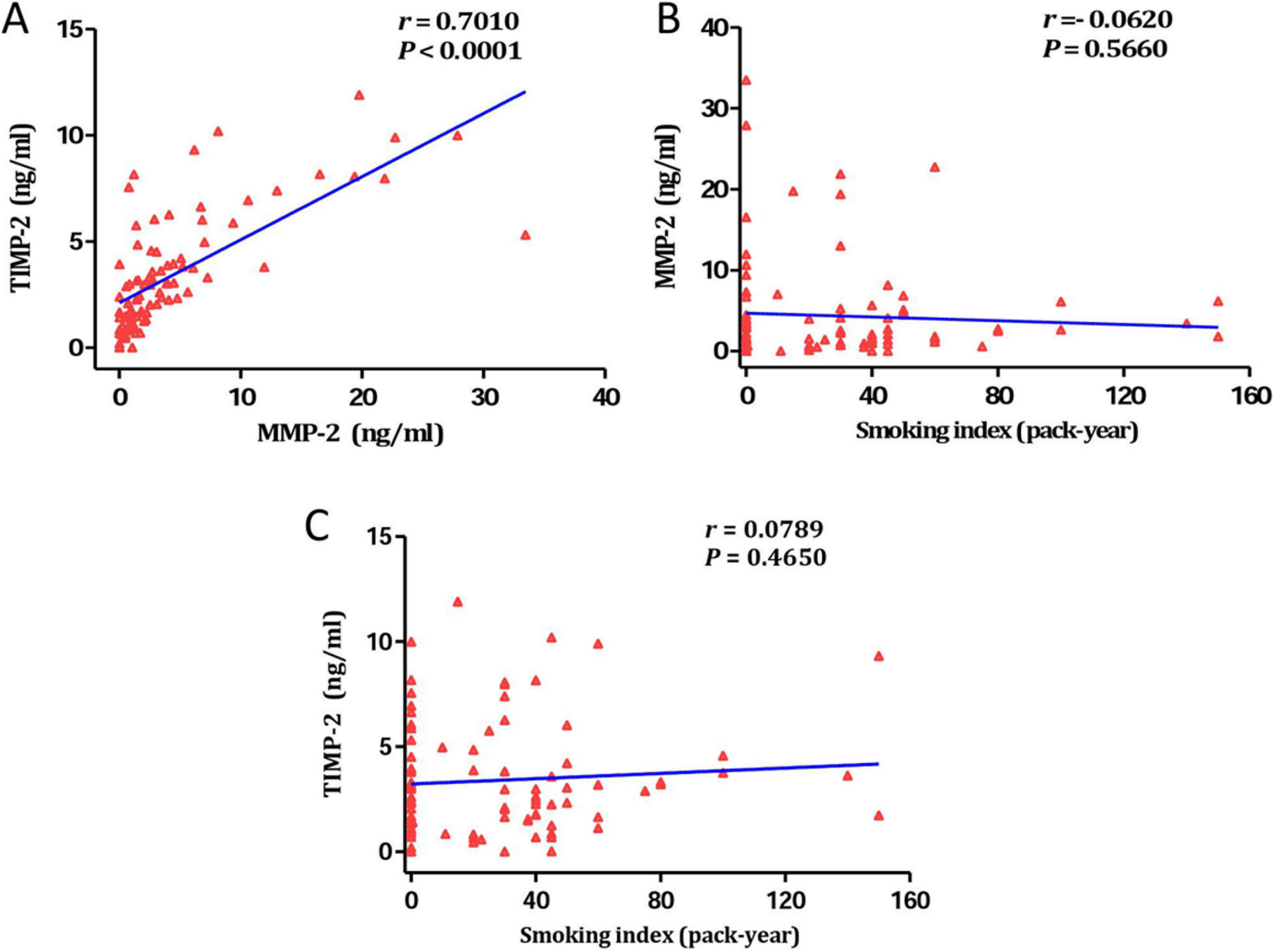
The relationship between smoking, MMP-2, and TIMP-2 (**A**) Statistically correlation was observed between levels of MMP-2 and TIMP-2 in BALF (*r* = 0.7010, *P* < 0.0001). (**B**) The levels of MMP-2 in BALF were not relevant to that of cigarette smoke (*r* = 0.0620, *P* = 0.5660). (**C**) No significant correlation was observed between cigarette smoke and TIMP-2 in BALF (*r* = 0.0789, *P* = 0.4650).

We evaluated the correlation of MMP-2, TIMP-2, and smoking index in BALF by the Pearson correlation analysis. The data showed a statistical significance correlation between the levels of MMP-2 and TIMP-2 in BALF, with a correlation coefficient (*r*) of 0.7010 (*P* < 0.001; Figure 3A). To determine how smoking behaved in MMP-2 and TIMP-2 expression, we made scatter plots of BALF MMP-2 and TIMP-2 levels and smoking index. Nevertheless, the levels of MMP-2 in BALF were not relevant to that of cigarette smoke (*r* = 0.0620, *P* = 0.5660; Figure 3B). Similarly, there was no significant correlation was observed between cigarette smoke and TIMP-2 in BALF (r = 0.0789, *P* = 0.4650; Figure 3C).

### Diagnostic value of BALF MMP-2 and TIMP-2 in patients with lung cancer

**Figure 4:**
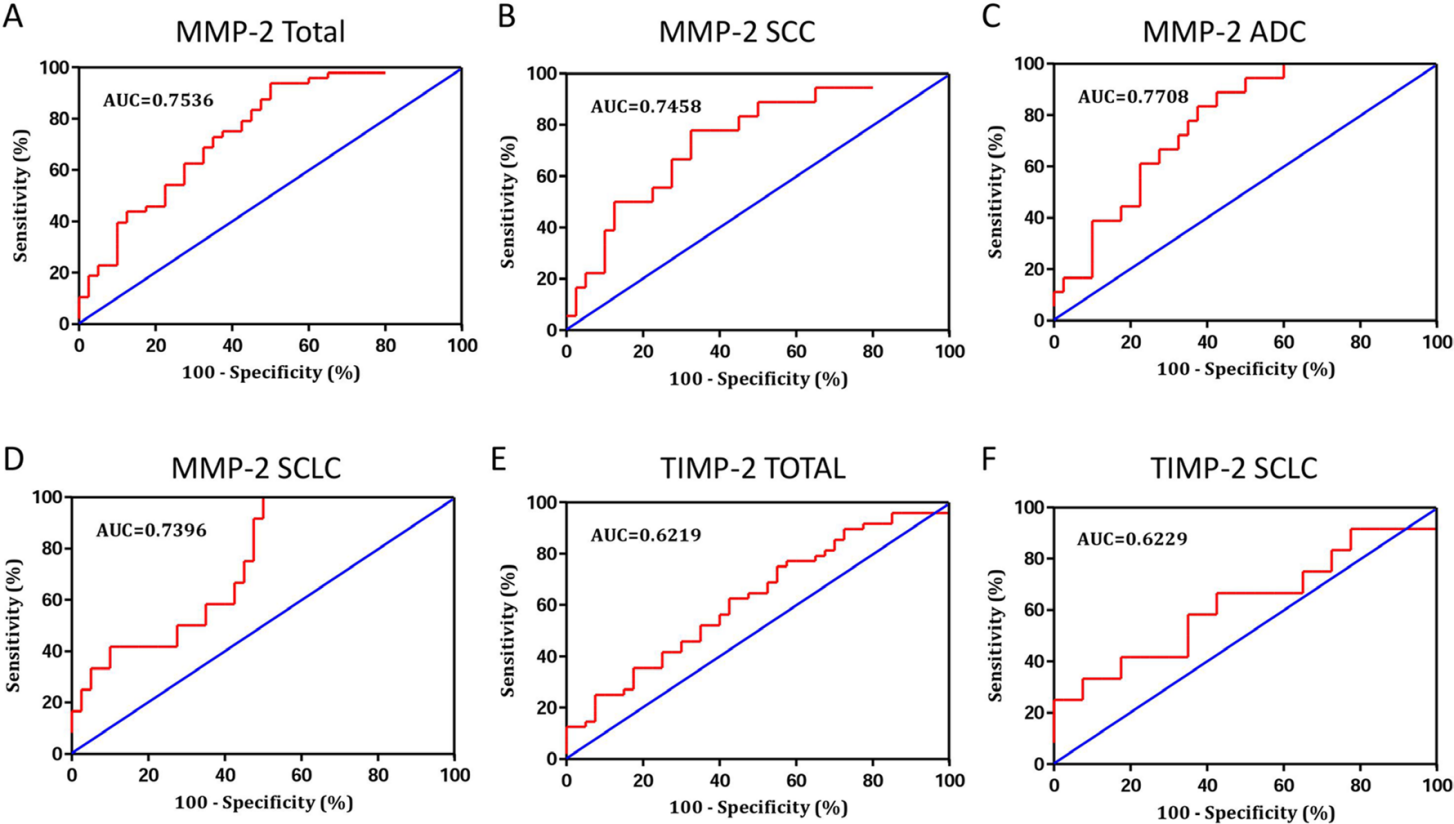
Receiver operating characteristic (ROC) curve was performed to evaluate the threshold value of MMP-2 and TIMP-2 in differentiating lung cancer from benign diseases (**A**) The levels of MMP-2 were robust in discriminating patients with lung cancer form benign diseases with an area under the curves (AUC) value of 0.7536 (95% CI, 0.6512–0.8561). (**B***–***D**) Comparing lung cancer patients with benign group, the AUC for SCC, ADC, and SCLC were 0.7458 (95% CI, 0.6097–0.8820), 0.7708 (95% CI, 0.6513–0.8904), and 0.7396 (95% CI, 0.5937–0.8854), respectively. (**E**) The area under the ROC for TIMP-2 was 0.6219 (95% CI, 0.5049–0.7388). (**F**) In subgroup patients with SCLC, TIMP-2 have an AUC of 0.6229 (95% CI, 0.4224–0.8234).

The present study has demonstrated that MMP-2 and TIMP-2 were useful biomarkers for lung cancer. Then, we assessed the usefulness of MMP-2 and TIMP-2 in BALF for differential diagnosis of pulmonary malignancy (Table 2). The diagnostic value of BALF MMP-2 for lung cancer was evaluated by Receiver operating characteristic (ROC) curves analysis. The results revealed that levels of MMP-2 were robust in discriminating patients with lung cancer form benign diseases with an area under the curves (AUC) value of 0.7536 (95% CI, 0.6512–0.8561) (Figure 4A). Using a cut-off value of 1.479 ng/ml, the sensitivity and specificity predictive values were 75.0% (95% CI, 60.4%–86.4%) and 62.5% (95% CI, 45.8%–77.3%), respectively, to identify a patient with lung cancer. To generate the optimum cutoff score, we further performed ROC curve by different cell type of lung cancer. Comparing lung cancer patients with benign group, the best cutoff level of MMP-2 in BALF for SCC, ADC, and SCLC were 1.794 ng/ml, 1.643 ng/ml, and 0.906 ng/ml, corresponding AUC was 0.7458 (95% CI, 0.6097–0.8820), 0.7708 (95% CI, 0.6513–0.8904), and 0.7396 (95% CI, 0.5937–0.8854), respectively (Figure 4B–4D). The diagnostic threshold afforded by the ROC analysis for TIMP-2 was 2.421 ng/ml. The area under the ROC was 0.6219 (95% CI, 0.5049–0.7388) (Figure 4E). With a threshold value of 2.421 ng/ml, TIMP-2 had a sensitivity of 62.5% (95% CI, 47.4%–76.1%), a specificity of 57.5% (95% CI, 40.9%–73.0%), in predicting the malignant nature of pulmonary mass. In subgroup patients with SCLC, TIMP-2 have an AUC of 0.6229 (95% CI, 0.4224–0.8234) (Figure 4F), with the sensitivity and specificity were 66.7% (95% CI, 34.9%–90.1%) and 57.5% (95% CI, 40.9%–73.0%), respectively.

**Table 2:** Diagnostic value of BALF MMP-2 and TIMP-2 in lung cancer

	AUC	Cut-off (ng/ml)	Sensitivity (%)	Specificity (%)	PPV (%)	NPV (%)
MMP-2						
Total	0.7536	1.479	75.0%	62.5%	70.1%	67.6%
SCC	0.7458	1.794	77.8%	67.5%	51.90%	87.1%
ADC	0.7708	1.643	77.8%	65.0%	50.00%	86.7%
SCLC	0.7396	0.906	100%	50.0%	37.5%	100%
TIMP-2						
Total	0.6219	2.421	62.5%	57.5%	63.8%	56.1%
SCLC	0.6229	2.421	66.7%	57.5%	32.0%	85.2%

BALF, bronchoalveolar lavage fluid; MMP-2, metal matrix proteinase 2; TIMP-2, tissue inhibitor of metalloproteinase 2; AUC, area under the curves; SCC, squamous cell carcinoma; ADC, adenocarcinoma; SCLC, small-cell lung cancer; PPV: positive predictive value; NPV: negative predictive value.

### Diagnostic accuracy of MMP-2 and TIMP-2 in the validation cohort

The validation cohort comprised 107 separate patients. The actual numbers of positive and negative diagnoses and several diagnostic accuracy measures using the cutoff from the derivation cohort were further tested on the validation dataset (Supplementary Table 2). Using a cut-off value of 1.479 ng/ml, the sensitivity, specificity, positive predictive value (PPV), and negative predictivevalue (NPV) for MMP-2 in the diagnosis of lung cancer were 75.0%, 62.5%, 70.1%, and 67.6%, respectively. Using a cut-off value of 2.421 ng/ml, the sensitivity, specificity, PPV, and NPV for TIMP-2 in the diagnosis of lung cancer were 62.5%, 57.5%, 63.8%, and 56.1%, respectively.

## DISCUSSION

The pulmonary mass is a common and challenging clinical problem. Distinguishing benign diseases from malignant is very important as to avoid patients undergoing surgery for a benign condition [8]. Recently, numerous studies have demonstrated that MMP-2 and TIMP-2 were involved in lung cancer development and prognosis [9–11]. However, most published studies detected those two markers in peripheral blood, which may confuse with other cancer situation and may not increase at an early stage. Changes of cytokines in airways directly reflect immunologic reactions of lung malignancies and increased much earlier and higher than that in peripheral blood [12, 13]. The utility of cytokines in airway for differential diagnosis of lung cancer has been described in several studies [14–19]. We therefore performed the present study to evaluate the expression of MMP-2 and TIMP-2 in airways of lung cancer patients by comparing levels of them in benign diseases. To our knowledge, this study is the first study to date that has assessed the MMP-2 and TIMP-2 in airways of lung cancer patients.

In this study, we found that the levels of MMP-2 was higher among patients with lung cancer than in patients with benign diseases. When the lung cancer cases were categorized by tumor histology, a statistically significant difference was found between patients with different cell type of lung cancer and benign diseases. Interesting, a statistical significance correlation was found between the levels of MMP-2 and TIMP-2 in BALF. A higher expression of TIMP-2 were also observed in airways of lung cancer patients than that of benign group. When the lung cancer cases were categorized by tumor histology, a statistically significant difference was also observed in patients with small cell carcinoma, while no significant difference was found between malignant and benign groups with respect to adenocarcinoma or squamous cell carcinoma.

Early detection of lung cancer improves survival rates, and towards this end, large screening methods in high-risk individuals have been suggested since the past century. Despite all efforts, there were still parts of patients were diagnosed at advance cancer, thus the need for novel or complementary lung cancer diagnostic and screening methods still exists. In this study, BALF MMP-2 and TIMP-2 concentration were not associated with TNM stage. We observed that BALF MMP-2 and TIMP-2 were increased at early stage lung cancer. These finding suggested that BALF MMP-2 and TIMP-2 might be serve as an early lung cancer biomarker.

ROC curve was further performed to evaluate the ability of MMP-2 in differentiating lung cancer patients from those with benign diseases. The diagnostic threshold afforded by the ROC analysis for MMP-2 was 1.479 ng/ml. With this cut-off value, MMP-2 had a sensitivity of 75.0% and a specificity of 62.5%, to identify a patient with lung cancer. To generate the optimum cutoff score, we further performed ROC curve by different cell type of lung cancer. Comparing lung cancer patients with benign group, the best cutoff level of MMP-2 in BALF for SCC, ADC, and SCLC were 1.794 ng/ml, 1.643 ng/ml, and 0.906 ng/ml, respectively. These findings were further confirmed by a validation cohort comprised 107 separate patients.

The diagnostic threshold afforded by the ROC analysis for TIMP-2 was 2.421 ng/ml, corresponding sensitivity and specificity were 62.5% and 57.5%, respectively. In subgroup patients with SCLC, with a cut-off value of 2.421 ng/ml, TIMP-2 had a sensitivity of 66.7% and a specificity of 57.5%, in predicting the malignant nature of pulmonary mass. However, BALF TIMP-2 was not significant increased among patients with adenocarcinoma or squamous cell carcinoma, thus the ROC analysis was not performed in those patients.

Some limitations of this study should be acknowledge. Firstly, the levels of MMP-2 and TIMP-2 in peripheral blood were not investigated. Secondly, the sensitivity and specificity of TIMP-2 for SCLC both less than 70%. Thus, the diagnostic value of TIMP-2 for SCLC is limited. Thirdly, all the controls in this study were patients with pulmonary diseases. The levels of MMP-2 and TIMP-2 in airways of health controls were still unclear. Fourthly, the biological mechanism of how MMP-2 and TIMP-2 modified the risk of lung cancer were not investigated in this study. Further studies on this context are wanted.

In conclusion, our study showed that the levels of MMP-2 and TIMP-2 were significantly higher in lung cancer patients than that of benign diseases. Most importantly, we observed that MMP-2 and TIMP-2 was increased in BALF even at early stage of lung cancer, implying that they might be served as early diagnosis biomarker. Measurement of MMP-2 and TIMP-2 in BALF might be helpful for differential diagnosis of primary lung cancer.

## MATERIALS AND METHODS

### Patients and specimens

The derivation cohort had a total of 88 patients who were found pulmonary mass by chest radiograph or CT screening. In validation cohort, 107 patients were enrolled. Approval for this study was obtained from the local ethics committee, and informed consent was obtained from all participating subjects. Clinical information regarding patient characteristics was based on patient records and registries. Furthermore, 20 lung tissue samples were collected for IHC study. All patients had histological confirmed and were excluded if they had received radiotherapy or chemotherapy. Smoking habits were defined at 1 year prior to diagnosis for cases or 1 year prior to interview for controls.

### Bronchoalveolar lavage (BAL)

The methods were described in details in our previous studies [13, 20, 21]. The bronchus with mass was washed with two 50-ml aliquots sterile physiological saline in 37°C. The fluid was gently withdrawn into a siliconized container placed in iced water. BALF was filtered through a nylon filter to remove mucus and centrifuged at 3,000 revolutions per minute for 10-min. The cell pellets were separated from the supernatants and stored at −80°C.

The lavage was done prior to brushing or biopsies to avoid contamination with blood. The bronchus on the disease side was washed with two 50-ml aliquots sterile physiological saline. The fluid was gently withdrawn into a siliconized container placed in iced water. The chilled lavage fluid was filtered through a nylon filter to remove mucus and centrifuged at 3,000 rpm for 10-min. The cell pellets were separated from the supernatants and stored at −80°C.

### ELISA

The levels of MMP-2 and TIMP-2 in BALF (ng/ml) were measured using Quantikine sandwich enzyme linked immunosorbent assays (ELISA; R&D systems, Minneapolis, MN, USA). The experimental procedures were followed in accordance with the rules designated by the manufacturer. Each sample was analyzed in duplicate, with dilutions as appropriate. The minimum detectable levels of MMP-2 and TIMP-2 were 0.5 ng/ml and 0.2 ng/ml, respectively.

### Immunohistochemistry

Paraffin-embedded tissues were cut into 4-μm-thick sections for dewaxing and rehydration. The tissue sections were incubated with primary antibody (anti-MMP-2 or anti-TIMP-2 mouse monoclonal antibody, 1:200, Abcam, Cambridge, UK) overnight at 4°C. The slides were washed three times in phosphate-buffered saline (PBS) and incubated with secondary antibody for 20 min. Staining slides were read on an Olympus optical microscope and scored according to the number of positively stained cells.

### Statistical analysis

Data was presented as means ± standard error of the mean (SEM). Comparison between different groups was done using the Student's *t*-test. The relationship between smoking index and MMP-2 and TIMP-2 levels was assessed by Pearson correlation. ROC curves were constructed to determine the diagnostic performance of MMP-2 and TIMP-2 levels in distinguishing malignant from benign pulmonary mass. The optimum cut-off point was determined as the value of the parameter that maximized the sum of specificity and sensitivity. The analyses were performed using GraphPad Prism 5.0 (GraphPad Software, San Diego, CA, USA), and all tests were two-sided with a significance level of 0.05.

## SUPPLEMENTARY MATERIALS TABLES


