# Supplementary material for: Elevated expression of MMP-2 and TIMP-2 cooperatively correlates with risk of lung cancer
Source: Oncotarget. 2017 Aug 11;8(46):80560–7. doi: 10.18632/oncotarget.20156 (PMC5655220; doi:10.18632/oncotarget.20156)
Supplement: Supplementary file 1 [file oncotarget-08-80560-s001.pdf]

Elevated expression of MMP-2 and TIMP-2 cooperatively correlates with risk of lung cancer

SUPPLEMENTARY MATERIALS

Supplementary Table 1: Clinical characteristics of patients in the validation cohort

	Lung cancer (*n* = 41)	Benign diseases (*n* = 66)	*P* value
Age, years			0.488
Mean ± SEM	60.2 ± 1.5	58.4 ± 1.3	
Gender, *n* (%)			0.122
Male	33 (80.5%)	44 (66.7%)	
Female	8 (19.5%)	22 (33.3%)	
Smoking status, *n* (%)			0.191
Smokers	27 (65.9%)	35 (53.0%)	
Non-smokers	14 (34.1%)	31 (47.0%)	
Cell type, *n* (%)			
Squamous cell carcinoma	19 (46.3%)		
Adenocarcinoma	14 (34.1%)		
Small-cell lung cancer	8 (19.5%)		

Supplementary Table 2: Diagnostic accuracy of MMP-2 and TIMP-2 in the validation cohort

	MMP-2	TIMP-2
Ture positive	33	29
False positive	17	29
True negative	49	37
False negative	8	12
Sensitivity	80.5%	70.7%
Specificity	74.2%	56.1%
Positive predictive value	66.0%	50.0%
Negative predictive value	86.0%	75.5%
